# Human Papillomavirus Vaccination Coverage Estimates Among the Primary Target Cohort (9–14-Year-Old Girls) in the World (2010–2024)

**DOI:** 10.3390/vaccines13101010

**Published:** 2025-09-27

**Authors:** Irena Ilic, Milena Ilic

**Affiliations:** 1 Faculty of Medicine, University of Belgrade, 11000 Belgrade, Serbia; 2 Department of Epidemiology, Faculty of Medical Sciences, University of Kragujevac, 34000 Kragujevac, Serbia

**Keywords:** human papillomavirus, cervical cancer, vaccine, coverage, trends

## Abstract

**Background/Objectives**: Monitoring human papillomavirus (HPV) vaccine coverage worldwide can provide valuable insight into cervical cancer prevention. The aim of this manuscript was to assess the HPV vaccination coverage among the primary target cohort (9–14-year-old girls) in the world from 2010 to 2024. **Methods**: A descriptive epidemiological study (with an ecological study design) was carried out. Trends in HPV vaccination coverage were examined using the joinpoint regression analysis. **Results**: The HPV vaccination was introduced into the national schedule of 147 countries in 2024. Globally, coverage with the first dose of the HPV vaccine in the primary target cohort (9–14-year-old girls) was estimated at 56.9% in 2024. The growth trend in HPV vaccination coverage was significant mainly in the most developed countries (e.g., such as the USA, Canada and Germany), while trends were 10 times faster in other countries such as Armenia, Indonesia and Tanzania. A decline in trends of HPV vaccination coverage was significant in some developing countries (e.g., such as Panama, Sri Lanka, and Suriname) and in one of the most developed countries—the United Kingdom. **Conclusions**: A better understanding of changes in HPV vaccination coverage worldwide and further efforts to improve coverage to the target of 90% may contribute to more effective disease prevention.

## 1. Introduction

Based on the most recent estimates of the Global Cancer Observatory (GLOBOCAN, provided by the International Agency for Research on Cancer of the World Health Organization, WHO), in 2022, cervical cancer ranked third in both incidence and mortality among women globally, with about 662,000 new cases (6.9% of the total) and 349,000 deaths (8.1% of the total) [1]. Out of that, around 196,000 (30%) of all incident cases in the world, as well as around 120,000 (34%) of total cervical cancer deaths, were reported in the South-East Asia region. Also, about 119,000 (18%) of all incident cases in the world, as well as about 77,000 (22%) of total cervical cancer deaths, were reported in the region of Africa. Cervical cancer ranks first in mortality among women in the region of Africa, also ranking second in incidence among women in the regions of Africa and South-East Asia. Women in Africa recorded the highest age-standardized rates for cervical cancer both in incidence (31.8 per 100,000) and mortality (21.4 per 100,000), followed by women in South-East Asia (17.7 and 10.8, respectively), while the lowest incidence and mortality rates were reported in the region of Europe (10.1 and 3.9, respectively). Differences in burden of cervical cancer can be explained by differences in socioeconomic development, as well as differences in lifestyle, habits, religious and cultural differences, comorbidities, development, availability of healthcare services, etc. [2]. However, the large burden of cervical cancer in low-resource countries is mainly a result of the absence of population screening programs [3,4]. If these trends continue, the annual number of new cases is projected to increase to 700,000 by 2030, while annual deaths will rise to 400,000 [5].

Numerous studies have shown favorable patterns and trends of incidence and mortality in cervical cancer among the elderly over the past decades, attributed to the implementation of organized screening [6,7,8]. In contrast to the trend of decreasing mortality, a trend of increasing incidence of cervical cancer has been observed in younger women (25–64 years) in recent decades [1,6,9,10]. Divergent trends in incidence and mortality from cervical cancer are particularly noticeable in women in the 25–44 age group in many of the most developed countries after the 1990s, whereby an increasing incidence trend emerged (Finland, The Netherlands, Sweden, Norway), or the incidence trend shows a plateau (the United States of America, Canada, Australia, Germany) [1]. While, in some countries, the decreasing trend in incidence follows the decreasing trend in mortality among younger women (Denmark, Spain), in other countries, the continuous trend of increasing incidence follows the trend of increasing mortality from cervical cancer (Japan). However, there were noticeable decreases in cervical cancer incidence rates in girls in the 15–19 and 20–24 age groups from 2007 to 2014, which could not be attributed to the implementation of programs of organized cervical screening either by the introduction of cervical cancer screening with the Papanicolaou (Pap) test or with Pap/Human papillomavirus (HPV) co-testing [11]. In 2021, about 31,000 new cases of cervical cancer occurred among women < 30 years worldwide [12].

While the overall trends in cervical cancer incidence and mortality declined, there still exists significant heterogeneity in the burden of cervical cancer at the global, regional and national level, primarily evident in the disproportionate level of risk among certain age categories of women. Therefore, additional efforts are needed to enhance prevention strategies aimed at reducing the burden of cervical cancer in the future [13,14].

Since the first HPV vaccine was licensed in mid-2006, routine HPV vaccination for women aged 11–12 has been recommended [15]. The majority of vaccines given until 2014 were quadrivalent vaccines, which target oncogenic HPV types 6, 11, 16, and 18 [16]. A 9-valent HPV vaccine became available in 2015, targeting the same types as the quadrivalent vaccine but with five additional oncogenic types (31, 33, 45, 52, and 58).

According to the “Global Strategy for cervical cancer elimination” adopted in 2020 by the World Health Assembly, a set of goals were developed for the 2020–2030 period that include scale-up of HPV vaccination (fully vaccinating 90% of girls with the HPV vaccine by the age of 15), screening for cervical cancer (screening 70% of women using a high-performance test by the age of 35 and again by the age of 45), and precancer and cancer treatment (treating 90% of women with precancer, and management of 90% of women with invasive cancer), with particular emphasis on low- and middle-income countries [17].

The emergence of the Coronavirus Disease 2019 (COVID-19) pandemic presented substantial obstacles to achieving the targets set out by the World Health Organization (WHO) and the United Nations’ Sustainable Development Goals (UNSDGs) in 2015 (i.e., a one-third reduction in premature mortality due to non-communicable diseases, such as cervical cancer, by 2030 through prevention and treatment) [18]. During the 2020–2021 period, 23 countries experienced a marked decline in HPV vaccination coverage (≥50% reduction) compared to 2019, with low- and middle-income countries being especially affected [19]. In addition, the global rollout of new national HPV immunization programs significantly reduced during the 2020–2021 period. The COVID-19 pandemic also led to a notable decrease in cervical cancer screening rates in many countries [20,21]. The above reflects the global inequity in cervical cancer prevention, where the disease burden is the highest in low- and middle-income countries, where community health facility access is limited and HPV vaccination, as well as HPV screening and treatment, is not widely implemented [4,21,22].

Monitoring HPV vaccine coverage can provide valuable insights into cervical cancer prevention and control [22,23]. A better understanding of the changes in HPV vaccination coverage worldwide can provide further insight into possible ways for more effective prevention of the disease [24].

The main goal of this manuscript is to describe first-dose HPV vaccine coverage among the primary target cohort (9–14-year-old girls) worldwide in 2024. Further, this study aimed to evaluate the trends regarding first-dose HPV vaccination coverage among the primary target cohort worldwide from 2010 to 2024. Also, this study aimed to examine the possible relationship between the global trends regarding first-dose HPV vaccination coverage and some measures of human development (including Gross Domestic Product (GDP), GDP per capita, the Human Development Index (HDI), and the Socio-development Index (SDI)) in the 2010–2024 period. Also, this study aimed to assess the correlation between national first-dose HPV vaccine coverage and measures of human development in 2024.

## 2. Materials and Methods

### 2.1. Study Design

This descriptive epidemiological study (with an ecological study design) included data of the annual estimates of HPV vaccine coverage to evaluate its trends in the world. This ecological study used aggregated global-, regional- and national-level data of HPV vaccination coverage, as well as aggregated (on population, i.e., global and national level) data on measures of human development. Hence, this ecological study is based on the comparison of groups or aggregates on population levels. Therefore, this study design allowed us to determine whether there is a correlation between the observed variables, but we were not able to determine whether there is a cause-and-effect relationship between them.

### 2.2. Data Sources

Data on HPV vaccine coverage estimates were extracted from the databases of the World Health Organization [25]. These WHO databases were compiled from 2010 to 2024, compiling epidemiological data for 194 countries, i.e., for all WHO member states. All WHO member states are obliged to report immunization coverage data to the WHO and the United Nations Children’s Fund (UNICEF) [26]. Countries officially report coverage data annually through the WHO/UNICEF Joint Reporting Form on Immunization.

The quality of HPV vaccination coverage estimates is determined by the quality and availability of data [27,28]. The HPV vaccination coverage estimates are based on government reports of vaccinations performed by healthcare providers (such as vaccination teams, physicians, health centers) at the administrative level, though sometimes surveys (such as household surveys, health survey, community surveys, etc.) are used. These methods provide accurate and reliable direct measures of coverage levels. However, these methods are also subject to biases (e.g., due to programmatic and other factors influencing immunization system performance—including vaccine shortages, changes in immunization policy, and civil unrest—or due to survey sample size). The WHO/UNICEF are continuously making efforts to improve the quality of data, coverage monitoring, methodologies for determining HPV vaccination coverage estimates [29].

Data for GDP and GDP per capita (in US dollars), as well as the HDI, were obtained from the United Nations data for National Accounts Main Aggregates database [30]. Data for the SDI were obtained from the Global Burden of Disease study [12].

### 2.3. Study Variables and Measures

HPV vaccine coverage is defined as the percentage (%) of the target population (girls) who received the recommended dose of HPV vaccine in a given year. Ensuring good HPV vaccine coverage involves delivering a first dose of the HPV vaccine to girls between the ages of 9 and 14 [25]. In this study, HPV vaccine coverage is presented at the global, regional (by 6 WHO regions—Africa, Americas, South-East Asia, Europe, Eastern Mediterranean, and Western Pacific), and national level.

The composite measure, which is the HDI, entails average achievement of key aspects of human development and well-being: life expectancy (health), literacy and school enrolments (education), and standard of living (income) [30].

The composite index, the SDI, comprises three indicators: income per capita, average educational attainment among persons ≥ 15 years, and fertility rate of females < 25 [12].

### 2.4. Statistical Analysis

Joinpoint regression analysis (Joinpoint regression software, Version 4.9.0.0—March 2021, made available via the Surveillance Research Program of the US National Cancer Institute), proposed by Kim et al. [31], was used to evaluate the temporal trends of HPV vaccination coverage. The so-called “joinpoints” are temporal points detected by joinpoint regression analysis when assessing magnitude and direction of trends, indicating that there was a significant change (increase or decrease) in HPV vaccination coverage. The Monte Carlo permutation test was used, with 4499 randomly selected datasets [31]. The Grid Search method was selected [32]. Analysis began with a minimum of zero joinpoints and tested whether a change in the trend was significant, up to the maximum of two (three segments) [31]. The Annual Percent Change (APC) and the Average Annual Percent Change (AAPC) were determined with a 95% confidence interval (95% CI) [33]. Terminology such as “significant increase” and “significant decrease” was used to depict trends (*p* < 0.05, based on statistical significance of APC/AAPC compared to zero). A “stable trend” corresponds with coverage remaining relatively constant over studied time, with no significant increases or decreases (APC/AAPC overlapped with zero, i.e., ≤0.5%; *p* > 0.5). For individual countries, this study only presents results based on the minimum number of joinpoints, even if there were changes in trends in the observed period. Analyses of trends involved only countries with data on HPV vaccine coverage available in the observed period continuously, provided that there were data continuously for at least 7 years in a row; countries with missing values in any year were excluded from the analysis.

A regression model was used to estimate the link between HPV vaccine coverage and GDP, GDP per capita, the HDI, and the SDI. The Pearson coefficient (r) was used to assess the correlation between HPV vaccine coverage and measures of human development.

For all tests, *p* < 0.05 was indicative of statistical significance.

### 2.5. Ethical Considerations

The study was conducted according to the guidelines of the Declaration of Helsinki and approved by the Ethics Committee of the Faculty of Medical Sciences, University of Kragujevac (Ref. No.: No. 01-14321). The study was conducted using publicly available data sources, based on fully aggregated (not individually identifiable) data.

## 3. Results

**Figure 1:**
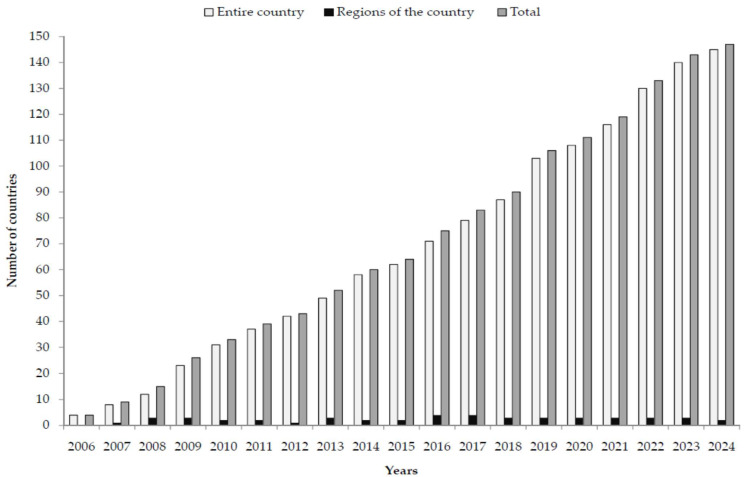
Number of countries with HPV vaccine in schedule (2006–2024). Source: World Health Organization estimates [25].

In 2006, when the first vaccine for the prevention of HPV-related disease was licensed, four countries introduced the HPV vaccine into their national vaccine schedule (Figure 1). By 2024, a total of 147 countries incorporated the HPV vaccine into their vaccination schedules, with 145 of them at the national level and 2 at the regional level.

**Figure 2:**
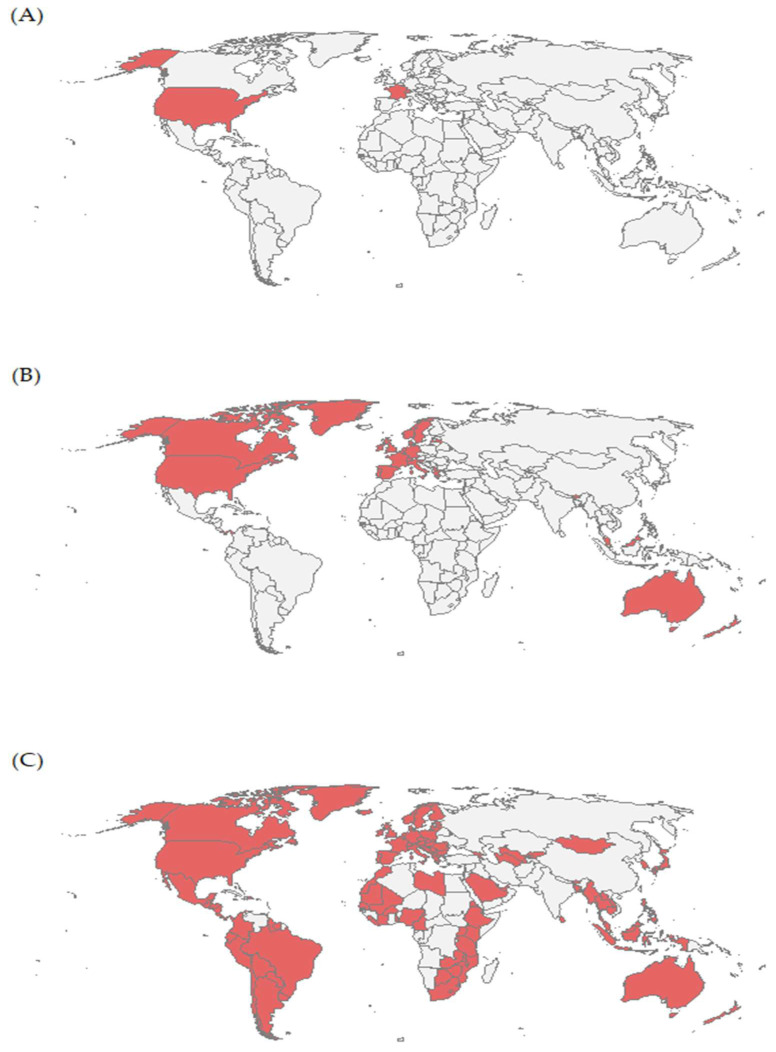
Number of countries with HPV vaccine in schedule in 2006 (**A**), in 2010 (**B**), and in 2024 (**C**). Source: World Health Organization estimates [25].

In 2006, all four countries that had introduced the HPV vaccine into their national vaccine schedules were high-income countries: the United States of America, France, Monaco, and Switzerland (Figure 2A). By 2010, another 30 countries (almost all of them being high-income countries) introduced the HPV vaccine into their vaccine schedules (Figure 2B). From 2011 to 2024, another 113 countries started using the HPV vaccine according to the national vaccination schedule (Figure 2C).

Globally, estimated HPV immunization coverage among the primary target cohort (9–14-year-old girls) in 2024 was 56.9% (Table 1). By WHO regions, the highest estimated HPV vaccination coverage in 2024 was 63.9% and was achieved in the South-East Asia Region. The lowest coverage (19.5%) was recorded in the Eastern Mediterranean Region.

**Figure 3:**
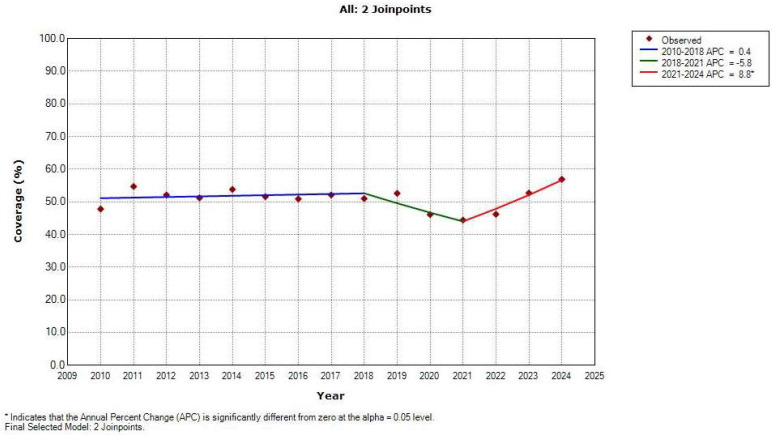
Trend in global human papillomavirus vaccine coverage (%) estimates among the primary target cohort (9–14-year-old girls) from 2010 to 2024: a joinpoint regression analysis. * statistically significant trend (*p* < 0.05). APC = Annual Percentage Change. Source: World Health Organization estimates [25].

From 2010 to 2024, the trend in global HPV vaccination coverage (%) estimates among the primary target cohort (9–14-year-old girls) non-significantly decreased (AAPC = −0.2; 95% CI = −1.1 to 0.7, *p* = 0.621) (Figure 3, Table 2). There were two joinpoints in the trend—coverage non-significantly increased by APC = 0.4% (95% CI = −1.1 to 1.9, *p* = 0.584) from 2010 to 2018, followed by a non-significant decrease by APC = −5.8% (95% CI = −17.7 to 7.9, *p* = 0.333) from 2018 to 2021, and then followed by a significant rise by +8.8% per year (95%CI = 1.7 to 16.4, *p* = 0.022) until the end of the period in 2024. The highest HPV vaccination coverage was achieved in 2024 (56.9%), and the lowest coverage was recorded in 2021 (44.4%).

**Figure 4:**
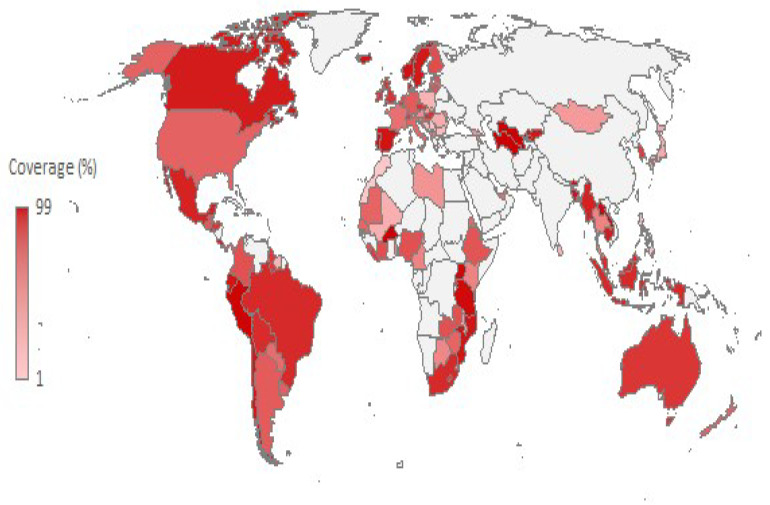
Human papillomavirus vaccination coverage (%) estimates * among the primary target cohort (9–14-year-old girls) in the world, by country, in 2024. * HPV coverage last year. Source: World Health Organization estimates [25].

The highest HPV vaccination coverage in 2024 (or in the year for which data is available) was >95%, achieved in Turkmenistan, Uganda, Timor-Leste, Burkina Faso, Peru, Uzbekistan, Cabo Verde, Lao People’s Democratic Republic, and Niue (Figure 4, Table 2). Coverage of 90–95% was recorded in Norway, Bhutan, Portugal, Spain, Tanzania, and Bangladesh. The lowest HPV vaccination coverage (<5%) was recorded in Serbia, Bahamas, Morocco, Antigua and Barbuda, and Qatar.

Table 2 provides a global overview of the implementation of HPV vaccination by WHO member states (including countries where HPV vaccination was introduced into the national schedule in 2010–2024, as well as countries where HPV vaccination was implemented but reports on vaccination coverage were not yet available; countries where HPV vaccination has not yet been implemented are also shown).

The growth trends in HPV vaccination coverage among the primary target cohort (9–14-year-old girls) from 2010 to 2024 were mainly significant in high-income countries, such as the United States of America (AAPC = 7.7; 95% CI = 6.2 to 9.4, *p* < 0.001), Canada (AAPC = 1.7; 95% CI = 1.1 to 2.2, *p* < 0.001), France (AAPC = 8.0; 95% CI = 4.2 to 11.9, *p* = 0.001), Germany (AAPC = 6.5; 95% CI = 5.7 to 7.2, *p* < 0.001), Norway (AAPC = 2.5; 95% CI = 1.7 to 3.3, *p* < 0.001), Israel (AAPC = 1.1; 95% CI = 0.3 to 1.9, *p* = 0.012), Sweden (AAPC = 2.8; 95% CI = 2.0 to 3.6, *p* < 0.001), Switzerland (AAPC = 5.2; 95% CI = 2.3 to 8.2, *p* = 0.002), Luxembourg (AAPC = 15.4; 95% CI = 10.6 to 20.4, *p* < 0.001), Andorra (AAPC = 8.1; 95% CI = 1.8 to 14.9, *p* = 0.021), Cyprus (AAPC = 6.8; 95% CI = 3.6 to 10.1, *p* = 0.003), and Finland, Belgium and Spain all experienced yearly increases of 1.6% (Table 2). Conversely, in some other countries (including Armenia, Austria, Croatia, Guyana, Indonesia, Palau, San Marino, the United Arab Emirates, and Tanzania) that reported lower HPV vaccination coverage (about 30–50%), trends were 10 times faster. Coverage levels of HPV vaccination of about 70–90% in some other high-income countries (including Australia, Bhutan, Czechia, Iceland, Latvia, Malta, Portugal, and Slovenia) were stable. Most countries that reported the highest HPV vaccination coverage, as well as a significant increasing trend in HPV vaccination coverage, had a school-based delivery strategy, while only a small number of countries with a favorable trend had facility-based health delivery or a mixed health system.

The declines in the trends regarding HPV vaccination coverage estimates among the primary target cohort (9–14-year-old girls) in the world from 2010 to 2024 were significant in some middle-income countries, such as Paraguay (AAPC = −10.8; 95% CI = −16.3 to −4.9, *p* = 0.003), Sri Lanka (AAPC = −21.1; 95% CI = −36.6 to −1.9, *p* = 0.038), and Suriname (AAPC = −26.3; 95% CI = −42.7 to −5.2, *p* = 0.023), and in one of the most developed countries—the United Kingdom (AAPC = −1.4; 95% CI = −2.6 to −0.2, *p* = 0.026) (Table 2).

**Figure 5:**
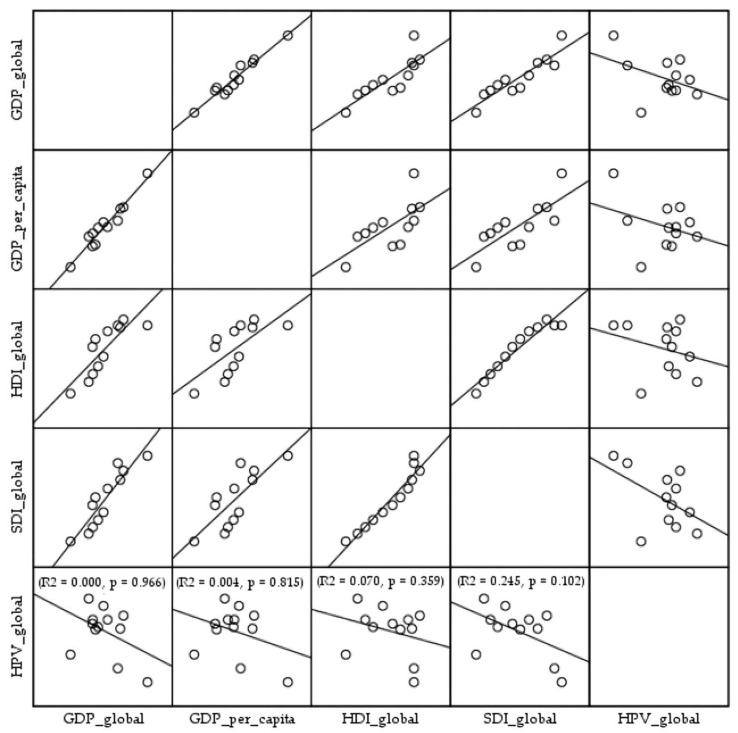
Association of HPV vaccination coverage estimates among the primary target cohort (9–14-year-old girls) worldwide with global Gross Domestic Product (GDP), GDP per capita, the Human Development Index (HDI), and the Sociodemographic Index (SDI), in 2010–2024. Sources: World Health Organization [25] and World Bank data [34].

The linear regression analyses showed absence of association between estimates of HPV vaccination coverage among the primary target cohort (9–14-year-old girls) worldwide, from 2010 to 2024, and global GDP, GDP per capita, and HDI and SDI (R2 = 0.000, *p* = 0.966; R2 = 0.004, *p* = 0.815; R2 = 0.070, *p* = 0.359; R2 = 0.245, *p* = 0.102, respectively) (Figure 5).

**Figure 6:**
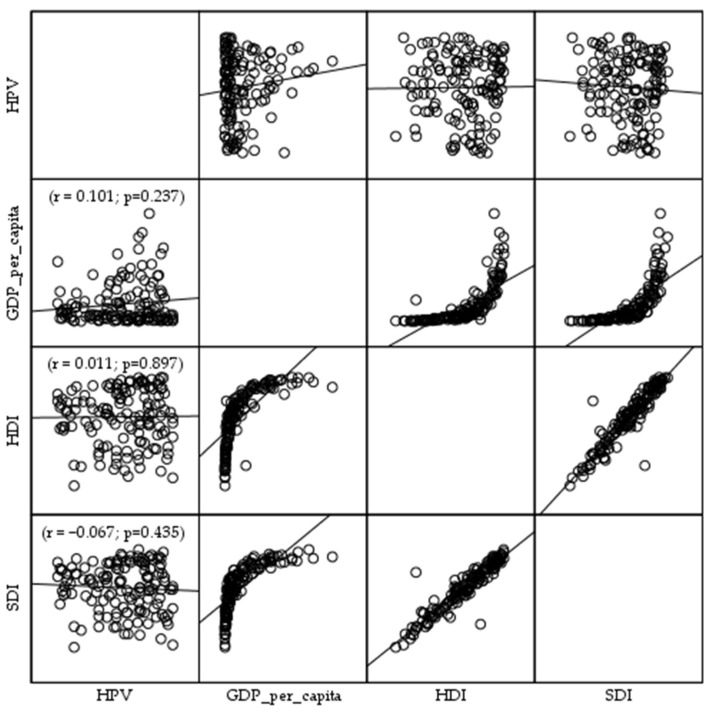
Correlation of the national HPV vaccination coverage estimates among the primary target cohort (9–14-year-old girls) with Gross Domestic Product per capita (GDP per capita), the Human Development Index (HDI), and the Sociodemographic Index (SDI) among countries in 2024. Sources: World Health Organization [25] and World Bank data [34].

The Pearson correlation coefficient showed absence of correlation between the national HPV vaccination coverage estimates among the primary target cohort (9–14-year-old girls) and GDP per capita (r = 0.101; *p* = 0.237), HDI (r = 0.011; *p* = 0.897), and SDI (r = −0.067; *p* = 0.435) among countries in 2024 (Figure 6).

## 4. Discussion

HPV vaccination was introduced into the national schedule of 147/194 WHO member states from 2006 to 2024. Estimates of HPV vaccination coverage vary considerably across countries, and global coverage remains significantly far below the target coverage of 90%. A slight decline in the estimated global HPV vaccination coverage among the primary target cohort (9–14-year-old girls) was observed in 2010–2024. Significant upward trends in HPV vaccination coverage were observed in some of the most developed countries. Significant downward trends in HPV vaccination coverage were observed in some developing countries (among them, as the only exception, was one of the most developed countries, the United Kingdom, where HPV vaccination coverage has been continuously declining).

Globally, compared to the HPV vaccination coverage among the primary target cohort (9–14-year-old girls) of 46.3% in 2010, in 2024, greater coverage was observed—56.9% (which, at the same time, is the highest global coverage of HPV vaccination on an annual level achieved so far). During the observed period, the lowest HPV vaccination coverage was reported in 2021 and was estimated at 44.4%. A recent systematic review which analyzed trends in HPV vaccination coverage from 2010 to 2023 reported that global-weighted average coverage of the first dose of HPV vaccine in girls aged 9–14 years was 61.6% in 2023 [35]. Compared to this systematic review, the slightly lower HPV vaccination coverage recorded in our study could be attributed to the use of only official WHO estimates for the analysis, as well as the inclusion of a smaller number of countries that introduced HPV vaccination in 2024 compared to previous years. However, data on HPV vaccination coverage is missing for about a quarter of WHO member states for 2024 (among them were mostly low-income countries but also several of the largest populations—such as India, China, Pakistan, Russian Federation, and Congo), which limits the conclusions that could be drawn from this study. However, regarding reports from recent years, first-dose HPV vaccination coverage among girls aged 9–14 in China in 2022 was 4% [36], and among women aged 15–49 in Vietnam, in 2021, the overall vaccination rate was 4% [37], although the HPV vaccine has not yet been introduced into the national calendars of these countries. Also, India is in the process of initially rolling out an HPV vaccination program, with a plan to expand it to the national level and establish routine vaccination for the primary target population of girls aged 9–14 years [38]. The findings of our study indicated that economic (GDP per capita) and development indicators (HDI, SDI) were unfavorable in countries not rolling out HPV vaccination as part of their national vaccination schedule (Afghanistan, Angola, Benin, Central African Republic, Chad, Guinea, Guinea-Bissau, Niger, Somalia, South Sudan, and Yemen). Some member states of the South-East Asia WHO Region (like Bangladesh and Bhutan) reported first-dose HPV vaccine coverage >90% in 2024, which could be due to their school-based delivery strategy, as well as to pilot/campaign programs in those populations, with support for their efforts by UNICEF, the WHO, the Global Alliance for Vaccines and Immunization—GAVI, and some international funds [22,25]. This finding could be due to the strategy of rolling out HPV vaccine in national immunization schedules, requiring the existence of an appropriate healthcare infrastructure that will ensure vaccine delivery, vaccine availability, and acceptance in the population, which still represents a challenge for the healthcare systems in low-income countries.

The HPV vaccination coverage estimates among the primary target cohort (9–14-year-old girls) vary remarkably across the world. Across 147 countries in 2024, both the highest HPV vaccination coverage (>90%) and the lowest HPV vaccination coverage (<5%) were observed mainly in low- and middle-income countries. In contrast, in recent years, the reported HPV vaccination coverage estimates in the most developed countries were mostly 50–90%. For example, some of the countries with the highest levels of social well-being indicators (GDP per capita, HDI, and SDI) at the same time have HPV vaccination coverage percentages of about 50–70% (the United States of America, Switzerland, Netherlands, New Zealand, Germany, Finland, France, and Italy). Apart from the issue of differences in the quality of data between countries, possible explanations for the large heterogeneity of estimated HPV vaccination coverage around the world in recent years include differences in capability and availability of public health institutions, the duration of HPV vaccination implementation in the national immunization schedule, funding, the procurement of vaccines, whether it was free or paid, whether it was mandatory or voluntary, migrations, the existence of nomadic populations, conflicts, etc. [39,40,41]. Even though some countries have reported >95% HPV vaccination coverage, such coverage values should be interpreted with caution, especially in countries with limited reporting capacity. This might reflect true high coverage in well-organized programs in some countries, but it might also represent an overestimation resulting from inconsistent administrative data. HPV vaccination hesitancy has also contributed to low uptake rates in some countries [42,43,44]. Expanded access through school-based programs has further contributed to higher HPV vaccine uptake in some countries (such as the United States of America, Canada, Australia, Belgium, the United Kingdom, and the Nordic countries) [40,45,46,47,48]. Also, the current study showed that a school-based vaccination delivery strategy was reported in most countries with a significant increase in HPV vaccination coverage and the highest coverage, while only a small number of countries with a favorable trend had facility-based health delivery or a mixed health system. A recent systematic literature review of HPV vaccination strategies regarding delivery systems within national immunization programs reported that school-based programs consistently reported achieving higher coverage than facility-only-based programs [49]. In addition, school-based vaccination programs are also associated with reduced socio-economic inequalities in HPV vaccination coverage, compared with vaccine delivery in health facilities [48,49,50]. However, despite high HPV vaccination coverage and school-based vaccination in high-income countries (such as Australia, Belgium, Canada, New Zealand, Norway, Sweden, Switzerland, and the United Kingdom), some studies reported associations between low HPV vaccination coverage with other sociodemographic factors, such as belonging to a minority ethnic group, being a migrant, parental education, and religion [51,52]. These studies have shown that, even in high-income countries with high HPV vaccination coverage, some inequalities should be further investigated, and the models needed to address them should be provided.

Considering the entire study period, global HPV vaccination coverage showed a non-significant decreasing trend, with two identified joinpoints—a non-significant increase from 2010 to 2018, followed by a non-significant decrease from 2018 to 2021, and then a significant increase until the end of the period in 2024. The first temporal segment, which is characterized by a non-significantly increasing global trend of the HPV vaccination coverage from 2010 to 2018, can be attributed to the increase in the number of countries that implemented the HPV vaccine in the national immunization schedule, increased the supply of low-cost vaccines, implemented school-based HPV vaccination programs, introduced supportive national recommendations and education campaigns, etc. [53,54,55]. The second temporal segment, which is characterized by a non-significantly decreasing global trend regarding the HPV vaccination coverage from 2018 to 2021, can be linked to delayed introductions of the HPV vaccine in national vaccination programs during the COVID-19 pandemic period (2020–2021) (globally, 17 countries implemented HPV vaccination in their national immunization schedule in 2019; in 2020, only four countries followed this step, and in 2021, there were only six countries), as well as reduced administration of routinely recommended HPV vaccines in a number of countries due to restriction measures, as a result of school closures; reluctance to seek health services due to the risk of spreading infection, redirecting time and the majority of resources to COVID-19 strategies of prevention and control in healthcare systems across the world; and a decrease in supply and access to immunization services, in addition to the spread of fake news or misinformation about vaccines safety, among other factors. [25,56,57,58]. The third temporal segment, which is characterized by a significantly increasing global trend in the HPV vaccination coverage from 2021 to 2024, can be attributed to the increase in the number of countries that implemented the HPV vaccine into the national immunization schedule (in 2022—14 countries; in 2023—13 countries), extending to the adoption of a single-dose HPV vaccine schedule with priority given to girls of the 9–14 age group, as recommended by the World Health Organization in 2022, introducing supportive national recommendations and education campaigns that could potentially further improve vaccination coverage across the world [59,60,61,62,63]. However, with respect to registered vaccination or under-reporting, the question always remains whether the changes in HPV vaccination coverage are real or partially reflect variations in data quality worldwide.

Among the countries, large heterogeneity in HPV vaccination coverage trends among the primary target cohort (9–14-year-old girls) was apparent. Favorable trends in HPV vaccination coverage were mainly observed in the most developed countries, while significant downward trends in HPV vaccination coverage were observed in some developing countries. Temporal differences in the start of implementing HPV vaccination into national immunization programs, in high initial prices of vaccines, in funding sources, in whether the HPV vaccine was offered free of charge or is not free, in vaccine availability, in vaccination delivery method (via school-based strategies and/or through healthcare facilities), and in parental education, as well as differences in the vaccination calendar across countries and vaccine procurement, may have impacted the magnitude and direction of vaccination coverage trends and affected the comparison between countries by introducing information bias. In addition, comparison of HPV vaccination coverage estimates is limited by changes in recommendations and the diversity of national HPV vaccination policies, with differences not only between countries but also within the same country over the years [29]. For example, although high-income countries were the first to introduce HPV vaccination (United States of America, France, and Spain) into national immunization calendars, due to the high initial price of the HPV vaccine, some low/lower-middle-income countries (Tanzania, Turkmenistan, Indonesia, Uganda, and Burkina Faso) that later implemented HPV vaccination quickly achieved greater coverage, which can be linked to pilot/campaign programs in those populations [22,25]. However, while many females in high/upper-middle-income countries have been vaccinated against HPV, many females in low-income countries characterized by the highest burden of cervical cancer still remain largely unprotected [22,64]. Towards improving HPV vaccination coverage, certain problems still exist, including the absence of registries to monitor individuals who have received vaccines or are in need of vaccines (which can lead to missed vaccination opportunities, incorrect registration and reporting, and, consequently, inaccurate coverage), financial burdens associated with healthcare and vaccine expenses, social inequities, limited healthcare accessibility in specific areas (the infrastructure issues, including supply shortages and staffing), disparities in state vaccination laws, inconsistency in guidelines, scarce health insurance coverage, insufficient dissemination of knowledge regarding vaccines, lack of trust, possible strategies to develop a positive attitude towards HPV vaccination, etc. [40,65,66,67]. The epidemiological impact of HPV vaccination on the pattern of HPV-related diseases has been confirmed by numerous studies, including those on the relationship between HPV vaccination and the subsequent reduced risk of invasive cervical cancer [68,69] and ecological studies that show that, with HPV vaccine introduction, cervical cancer incidence rates declined [70,71]. Accordingly, concomitant HPV vaccination and screening seem to be realistic options for elimination of cervical cancer [72,73].

Although HPV vaccines have been available for 20 years, global HPV vaccination coverage in 2024 is still low and remains far below the WHO target of 90% of girls fully vaccinated with the HPV vaccine by the age of 15. In addition to contributing to the expansion of global vaccine uptake and coverage by simplifying implementation and significantly reducing costs, single-dose HPV vaccination produces an immune response and protection against HPV infection [74,75,76]. Numerous studies suggest that one dose of the HPV vaccine has comparable effectiveness to two or three doses in prevention of cancerous cervical lesions, and studies also show that providing one dose of the HPV vaccine may be a sustainable strategy towards the global elimination of cervical cancer [17,25,29,59,74,75,76]. To achieve the WHO target of 90% vaccination coverage by 2030, as well as increase the number of countries that have introduced HPV vaccination into national immunization programs, large investments are needed around the world in the coming years [77,78,79]. Along with continued implementation of the HPV vaccine in the national immunization schedules, monitoring of HPV vaccination coverage is fundamental to assess the performance of vaccination programs and health systems, as well as the potential impact of HPV vaccination on elimination and eradication of HPV-related diseases.

### The Strengths and Limitations of the Study

This study evaluated global patterns and trends in HPV vaccination coverage from 2010 to 2024 for the world’s primary target cohort, i.e., girls aged 9–14 years. Further, this study reported estimates of HPV vaccination coverage for 147 WHO member states, using data from the WHO/UNICEF database. WHO/UNICEF estimates are the key source of data on vaccination at the national level and represent the largest global source of data on HPV immunization coverage in a standardized manner. Furthermore, by including the latest available data in this analysis, this study provides estimates of HPV vaccination coverage for the period of the COVID-19 pandemic. In addition, the joinpoint regression analysis provides an overview of the magnitude and direction of changes in time trends and determines whether these changes are statistically significant. Finally, this study analyzed the correlation of global patterns and trends in HPV vaccination coverage with economic (GDP, GDP per capita) and development indicators (HDI, SDI).

However, there are some sources of limitations to this study that should be considered. First of all, the question of quality of HPV vaccination coverage data can always be raised (including availability, timeliness, data accuracy, administrative coverage, completeness of reporting, estimation of target population size, standardization techniques used, etc.). Potential sources of data limitations include variations in record-keeping practices, reporting bias that may vary depending on the quality of health services, the development of health infrastructure, and heterogeneity of vaccination strategies across countries, which may affect the reliability of cross-country comparison. Also, due to the issue of under-reporting of HPV vaccination data, especially in developing countries, the presence of bias cannot be completely eliminated. In addition, the important limitation of this analysis was missing data, either regarding the absence of reporting as a whole or the absence of data for individual annual reports as a whole, as well as reports that marked annual HPV vaccination coverage as 0%. Therefore, it was not possible to conduct a joinpoint regression analysis of temporal trends for HPV vaccination coverage worldwide due to data limitations in some countries. Consequently, the findings that resulted from the comparison across countries should be interpreted very carefully. Also, the absence of data on population characteristics (such as race, ethnicity, parental education, place of residence, etc.) limited the analysis of the effects of these factors on HPV vaccination coverage. Further, another limitation of this study is ecological fallacy, i.e., fallacy inherent to this study design, as a consequence of drawing conclusions about associations based on population-level data due to missing data at the individual level (e.g., for socioeconomic status, occupation, employment, sexual activity, comorbidities). Finally, our analysis considered only first-dose coverage, which can be considered a valuable indicator of HPV immunization program initiation and access. Despite these limitations, this study provides useful insights into the variations and inequalities in the patterns and trends regarding HPV vaccination coverage worldwide and could provide help for health authorities and policy makers to develop more effective prevention strategies based on reliable estimates of vaccination coverage.

Finally, the findings of this study must be further elucidated in analytical longitudinal studies that would be based on more complete data from a larger number of countries during a longer period of monitoring the implementation of HPV vaccination.

## 5. Conclusions

There are large international differences in the patterns and temporal trends of HPV vaccination coverage estimates among the primary target cohort (9–14-year-old girls) in the world. Globally, 147/194 WHO member states introduced HPV vaccination in national immunization schedules between 2006 and 2024, but vaccination coverage was very heterogeneous across countries. Global HPV vaccination coverage (56.9%) in 2024 remains far below the WHO target of 90% coverage for girls aged 9 to 14 years. The growth trends in the HPV vaccination coverage were significant mainly in the most developed countries, while the declining trends of vaccination coverage were significant in some developing countries. A better understanding of changes in HPV vaccination coverage worldwide and further efforts to improve coverage among the primary target cohort (9–14-year-old girls) may contribute to more effective disease prevention. Future efforts for improving HPV vaccination coverage globally should consider the role of expanding school programs, addressing hesitancy, ensuring sustainable financing, and learning from successful experiences.

## Figures and Tables

**Table 1:** HPV vaccination coverage estimates among the primary target cohort (9–14-year-old girls) (%), by World Health Organization regions, in 2024.

	Global	WHO Region					
		African Region	Region of the Americas	Eastern Mediterranean Region	European Region	South-East Asia Region	Western Pacific Region
Countries with HPV vaccination in schedule, no. (%)	147/194 (75.8)	29/47 (61.7)	32/35 (91.4)	8/21 (38.1)	47/53 (88.7)	7/11 (63.6)	24/27 (88.9)
HPV vaccination coverage (%)	56.9	57.9	55.4	19.5	55.6	63.9	58.2

**Table 2:** Human papillomavirus vaccination coverage (%) estimates among the primary target cohort (9–14-year-old girls) in the world—by location, income level, and delivery strategy—in the 2010–2024 period; a joinpoint regression analysis was performed *.

Locations	2010/ First Available	2024/ Last Available	AAPC (95%CI)	*p* Value	Income Group ***	Delivery Strategy
Global	47.8	56.9	−0.2 (−1.1 to 0.7)	0.621		
Countries **						
Afghanistan					LIC	
Albania	18	43	-	-	UMIC	Facility-based
Algeria					UMIC	
Andorra	49	82	8.1 * (1.8 to 14.9)	0.021	HIC	School-based
Angola					LMIC	
Antigua and Barbuda	2	1	-	-	HIC	Facility-based
Argentina	36	55	0.44 (−2.2 to 3.1)	0.725	UMIC	Mixed
Armenia	2	31	48.0 * (27.9 to 71.2)	<0.001	UMIC	Facility-based
Australia	67	73	0.4 (−0.6 to 1.5)	0.397	HIC	School-based
Austria	1	31	54.4 * (21.6 to 95.9)	0.003	HIC	School-based
Azerbaijan					UMIC	
Bahamas	1	3	−6.7 (−23.7 to 14.0)	0.447	HIC	School-based
Bahrain	/	74	-	-	HIC	Not available
Bangladesh	19	90	-	-	LMIC	Not available
Barbados	8	43	9.3 (3.9 to 24.2)	0.152	HIC	School-based
Belarus					UMIC	
Belgium	59	72	1.6 * (1.3 to 1.8)	<0.001	HIC	School-based
Belize	51	62	−5.6 (−37.9 to 43.4)	0.747	UMIC	School-based
Benin					LMIC	
Bhutan	96	92	0.3 (−1.3 to 1.9)	0.715	LMIC	School-based
Bolivia	65	78	1.4 (−14.5 to 20.2)	0.849	LMIC	School-based
Bosnia and Herzegovina	5	9	-	-	UMIC	Mixed
Botswana	65	34	-	-	UMIC	School-based
Brazil	61	79	2.2 (−1.1 to 5.5)	0.169	UMIC	Facility-based
Brunei Darussalam	88	88	0.7 (−0.1 to 1.4)	0.066	HIC	Mixed
Bulgaria	21	9	−8.7 (−19.8 to 3.9)	0.147	HIC	Facility-based
Burkina Faso	16	99	-	-	LIC	School-based
Burundi					LIC	
Cabo Verde	99	99	-	-	UMIC	Not available
Cambodia	87	85	-	-	LMIC	Not available
Cameroon	5	36	-	-	LMIC	School-based
Canada	73	86	1.7 * (1.1 to 2.2)	<0.001	HIC	School-based
Central African Republic					LIC	
Chad					LIC	
Chile	76	87	0.2 (−2.8 to 3.3)	0.883	HIC	School-based
China					UMIC	
Colombia	86	60	1.3 (−16.7 to 23.3)	0.886	UMIC	School-based
Comoros					LMIC	
Congo, Republic of the					LMIC	
Cook Islands	74	37	−8.3 (−17.3 to 1.7)	0.092		School-based
Costa Rica	56	84	−2.8 (−21.3 to 20.2)	0.730	HIC	School-based
Côte d’Ivoire	12	61	-	-	LMIC	School-based
Croatia	3	53	40.4 * (23.1 to 60.1)	<0.001	HIC	School-based
Cuba					UMIC	
Cyprus	57	89	6.8 * (3.6 to 10.1)	0.003	HIC	School-based
Czechia	74	75	0.2 (−0.8 to 1.3)	0.628	HIC	Facility-based
Korea (South)					HIC	
DR Congo					LIC	
Denmark	68	81	2.6 (−3.8 to 9.5)	0.402	HIC	Facility-based
Djibouti					LMIC	
Dominica	74	77	-	-	UMIC	School-based
Dominican Republic	34	45	8.2 (−26.0 to 58.4)	0.629	UMIC	Facility-based
Ecuador	81	89	−10.5 (−29.5 to 13.6)	0.319	UMIC	School-based
Egypt					LMIC	
El Salvador	24	86	-	-	UMIC	School-based
Equatorial Guinea					UMIC	
Eritrea	84	49	-	-	LIC	Not available
Estonia	46	63	1.8 (−5.0 to 9.0)	0.533	HIC	School-based
Eswatini	61	30	-	-	LMIC	School-based
Ethiopia	24	58	-	-	LIC	School-based
Fiji	53	71	0.4 (−3.2 to 4.1)	0.812	UMIC	School-based
Finland	55	62	1.6 * (0.2 to 3.0)	0.034	HIC	School-based
France	25	45	8.0 * (4.2 to 11.9)	0.001	HIC	Facility-based
Gabon					UMIC	
Gambia	24	15	-	-	LIC	School-based
Georgia	11	29	-	-	UMIC	Facility-based
Germany	27	55	6.5 * (5.7 to 7.2)	<0.001	HIC	Facility-based
Ghana					LMIC	
Greece					HIC	
Grenada	42	5	-	-	UMIC	School-based
Greenland					HIC	
Guatemala	31	57	-	-	UMIC	School-based
Guinea					LMIC	
Guinea-Bissau					LIC	
Guyana	2	71	18.9 * (0.7 to 40.4)		HIC	School-based
Haiti					LMIC	
Honduras	52	72	1.5 (−1.8 to 4.9)	0.310	LMIC	School-based
Hungary	75	75	0.6 (−0.5 to 1.8)	0.241	HIC	School-based
Iceland	92	89	−0.2 (−0.8 to 0.3)	0.328	HIC	School-based
India					LMIC	
Indonesia	3	79	58.4 * (14.6 to 118.9)	0.013	HIC	School-based
Iran					UMIC	
Iraq					UMIC	
Ireland	82	73	−1.0 (−2.8 to 0.9)	0.263	HIC	School-based
Israel	51	58	1.1 * (0.3 to 1.9)	0.012	HIC	School-based
Italy	44	51	−2.1 (−4.6 to 0.5)	0.102	HIC	Mixed
Jamaica	8	7	−8.3 (−33.9 to 27.1)	0.526	UMIC	School-based
Japan	12	17	-	-	HIC	Facility-based
Jordan					LMIC	
Kazakhstan	/	38	-	-	UMIC	School-based
Kenya	16	36	-	-	LMIC	Facility-based
Kiribati	55	71	-	-	LMIC	Not available
Kuwait					HIC	
Kyrgyzstan	48	88	-	-	LMIC	School-based
Lao People’s Republic	54	95	-	-	LMIC	School-based
Latvia	58	51	−0.5 (−3.7 to 2.8)	0.721	HIC	Facility-based
Lebanon					LMIC	
Lesotho	55	70	-	-	LMIC	Not available
Liberia	17	71	-	-	LIC	Mixed
Libya	30	28	-	-	UMIC	Not available
Lithuania	28	59	4.2 (−7.3 to 17.0)	0.425	HIC	Facility-based
Luxembourg	16	79	15.4 * (10.6 to 20.4)	<0.001	HIC	Facility-based
Madagascar					LIC	
Malawi	74	21	-	-	LIC	School-based
Malaysia	83	78	-	-	UMIC	School-based
Maldives	66	55	-	-	UMIC	School-based
Mali	/	15	-	-	LIC	Mixed
Malta	93	80	−0.1 (−1.1. to 0.9)	0.852	HIC	Facility-based
Marshall Islands	37	50	7.2 (−1.1 to 16.2)	0.086	UMIC	Mixed
Mauritania	24	51	-	-	LMIC	Not available
Mauritius	74	92	−9.0 (−25.4 to 11.0)	0.288	UMIC	School-based
Mexico	82	82	−11.0 (−29.3 to 12.1)	0.291	UMIC	School-based
Micronesia	32	40	−6.4 (−14.7 to 2.8)	0.137	LMIC	School-based
Monaco	/	14	-	-	HIC	Facility-based
Mongolia	/	25	-	-	UMIC	School-based
Montenegro	18	5	-	-	UMIC	Facility-based
Morocco	/	3	-	-	LMIC	Not available
Mozambique	32	89	-	-	LIC	School-based
Myanmar	44	83	-	-	LMIC	School-based
Namibia					LMIC	
Nauru	40	6	-	-	HIC	Not available
Nepal					LMIC	
Netherlands	52	63	0.8 (−0.6 to 2.3)	0.241	HIC	Facility-based
New Zealand	45	52	1.3 (−0.1 to 2.7)	0.070	HIC	Mixed
Nicaragua					LMIC	
Niger					LIC	
Nigeria	27	60	-	-	LMIC	Mixed
Niue	76	99	-	-		School-based
North Macedonia	31	40	0.7 (−2.4 to 3.8)	0.650	UMIC	School-based
Norway	58	91	2.5 * (1.7 to 3.3)	<0.001	HIC	School-based
Oman					HIC	
Pakistan					LMIC	
Palau	9	43	18.7 * (12.4 to 25.4)	<0.001	HIC	School-based
Panama	65	54	−2.1 * (−4.2 to −0.0)	0.048	HIC	Mixed
Papua New Guinea					LMIC	
Paraguay	79	47	−10.8 * (−16.3 to −4.9)	0.003	UMIC	Mixed
Peru	64	97	-	-	UMIC	School-based
Philippines	3	5	-	-	LMIC	School-based
Poland	2	13	-	-	HIC	Facility-based
Portugal	88	92	0.2 (−0.6 to 1.0)	0.596	HIC	Facility-based
Qatar	/	1	-	-	HIC	Not available
Republic of Korea	50	69	2.1 (−2.3 to 6.7)	0.296	LIC	Facility-based
Republic of Moldova	47	42	0.3 (−5.1 to 6.0)	0.893	UMIC	School-based
Romania	23	17	-	-	HIC	Facility-based
Russian Federation					HIC	
Rwanda	34	69	-	-	LIC	School-based
Saint Kitts and Nevis	98	78	-	-	HIC	School-based
Saint Lucia	42	71	-	-	UMIC	School-based
Saint Vincent/Grenadines	4	8	-	-	UMIC	School-based
Samoa	87	64	-	-	UMIC	School-based
San Marino	21	56	13.3 * (6.4 to 20.5)	0.001	HIC	Facility-based
Sao Tome and Principe	57	75	-	-	LMIC	Not available
Saudi Arabia	47	/	-	-	HIC	School-based
Senegal	26	51	-	-	LMIC	Facility-based
Serbia	0	4	-	-	UMIC	Not available
Seychelles	77	23	−9.7 * (−17.9 to −0.8)	0.036	HIC	School-based
Sierra Leone	24	61	-	-	LIC	Not available
Singapore	1	70	-	-	HIC	School-based
Slovakia	29	24	-	-	HIC	Not available
Slovenia	44	43	0.2 (−1.4 to 1.8)	0.801	HIC	School-based
Solomon Islands	17	78	-	-	LMIC	Mixed
Somalia					LIC	
South Africa	66	79	-	-	UMIC	School-based
South Sudan					LIC	
Spain	61	90	1.6 * (0.7 to 2.6)	0.003	HIC	Mixed
Sri Lanka	12	12	−21.1 * (−36.6 to −1.9)	0.038	LMIC	Mixed
Sudan					LIC	
Suriname	36	13	−26.3 * (−42.7 to −5.2)	0.023	UMIC	School-based
Sweden	73	87	2.8 * (2.0 to 3.6)	<0.001	HIC	School-based
Switzerland	20	70	5.2 * (2.3 to 8.2)	0.002	HIC	Mixed
Syrian Arab Republic					LIC	
Tajikistan					LMIC	
Thailand	66	36	-	-	UMIC	School-based
Timor-Leste	/	99	-	-	LMIC	School-based
Togo	45	36	-	-	LIC	School-based
Tonga	12	67	-	-	UMIC	Not available
Trinidad and Tobago	8	16	5.2 (−1.1 to 12.0)	0.097	HIC	Mixed
Tunisia					LMIC	
Türkiye					UMIC	
Turkmenistan	90	99	0.9 (−0.0 to 1.9)	0.055	UMIC	Mixed
Tuvalu	27	70	-	-	UMIC	Not available
Uganda	30	95	7.4 (−6.2 to 23.1)	0.253	LIC	School-based
Ukraine					UMIC	
United Arab Emirates	25	46	9.3 * (5.2 to 13.6)	0.002	HIC	School-based
United Kingdom	77	75	−1.4 * (−2.6 to −0.2)	0.026	HIC	School-based
Tanzania	19	94	24.9 * (10.3 to 41.4)	0.006	LMIC	Mixed
United States of America	23	52	7.7 * (6.2 to 9.4)	<0.001	HIC	Facility-based
Uruguay	26	54	3.5 (−0.9 to 8.2)	0.106	HIC	Facility-based
Uzbekistan	97	99	-	-	LMIC	School-based
Vanuatu	45	43	-	-	LMIC	School-based
Venezuela					UMIC	
Vietnam					LMIC	
Yemen					LIC	
Zambia	68	60	-	-	LMIC	School-based
Zimbabwe	64	51	-	-	LMIC	Mixed

* Statistically significant trend (*p* < 0.05, based on statistical significance of Average Annual Percent Change = AAPC compared to zero). ** results of joinpoint regression analyses are not shown for HPV vaccination coverage in some countries, either because of absence of data (either because HPV vaccination has not yet been introduced or because data on HPV vaccination coverage have not yet been reported; marked in gray) or because there was no data for the HPV vaccination coverage in the whole observed period continuously (marked as “/”). For joinpoint regression analyses of trends, we present all data available from 2010 onwards, provided that there were data for at least 7 consecutive years in a row continuously. Note: if the data were not continuous or the data included 0 as percentage of vaccination coverage, the joinpoint regression software did not allow analyses of trends (marked as “-”). Source: World Health Organization [25]. *** countries by available World Bank income group for 2024 (low-income countries—LICs (USD 1135 or less), lower-middle-income countries—LMICs (USD 1136 to USD 4495), upper-middle-income countries—UMICs (USD 4496 to USD 13,935), and high-income countries—HICs (USD 13,936 or more). Data for Cook Islands and Niue were not available. Source: World Bank data [34].

## Data Availability

The original contributions of this study are included in the article.
